# Supplementary material for: Improvement of On-Site Sensor for Simultaneous Determination of Phosphate, Silicic Acid, Nitrate plus Nitrite in Seawater
Source: Sensors (Basel). 2022 May 3;22(9):3479. doi: 10.3390/s22093479 (PMC9104159; doi:10.3390/s22093479)
Supplement: Supplementary file 1 [file sensors-22-03479-s001.zip › sensors-1664971-supplementary.pdf]

sensors

MDPI

*Supplementary Material*

# Improvement of *on-site* sensor for simultaneous determination of phosphate, silicic acid, nitrate and nitrite in seawater

**Mahmoud Fatehy Altahan [1,2,*], Mario Esposito [1] and Eric P. Achterberg [1,*]**

[1] GEOMAR Helmholtz Centre for Ocean Research Kiel, 24148 Kiel, Germany
[2] Central Laboratory for Environmental Quality Monitoring, National Water Research Center, 13621 El-Qanater El-Khairia, Egypt

* Correspondence: maltahan@geomar.de, mahmoud_abdalqader@nwrc.gov.eg (M.F.Altahan), eachter-berg@geomar.de (E.P. Achterberg)

**Table S1.** Detailed Procedure for measurements during the analyzer deployment with the number of ports as mentioned in **Figure 2.**

| | | Step | | Port open | Linear movement[a] | | Operation & Description |
|---|---|---|---|---|---|---|---|
| | | 1 | a | 3 | 4500 (r[b]) | | Draw up the cleaning solution into the syringe and all detectors, and set the light intensity for blank measurement. |
| | | | b | 8 | 4500 (i[c]) | | |
| | | | c | 3 | 4500 (r) | | |
| | | | d | 15 | 4500 (i) | | |
| | | | e | 3 | 4500 (r) | | |
| | | | f | 16 | 4500 (i) | | |
| | | 2 | | 4 | 4500 (i) | | Initialize the syringe motor and detector, align the switching valve and ensure that the syringe plunger is fully retracted. |
| $PO_4^{3-}$ Calibration | Blank | 3 | a | 7 | 4500 (r) | 2x flushing | Washing the syringe and the $PO_4^{3-}$ detector with deionized water |
| | | | b | 8 | 4500 (i) | | |
| | | 4 | a | 7 | 2700 (r) | | Draw up the blank and set the light intensity for the reference $(I^R, V^R)$ |
| | | | b | 4 | 1350 (i) | | |
| | | | c | 8 | 1350 (i) | | |
| | | 5 | a | 8 | 720 (r) | | Withdraw the blank from the flow cell and add R1 (Molybdate mixed reagent) and R2 (Ascorbic Acid) |
| | | | b | 9 | 180 (r) | | |
| | | | c | 10 | 180 (r) | | |
| | | 6 | a | 8 | 450 (i) | 4x mixing | Mixing the reagent with the blank and inject the reaction mixture inside the detector and waiting for 3 minutes for color development and set the light intensity for the measurement $(V,\ I)$ |
| | | | | | 450 (r) | | |
| | | | b | 4 | 180 (i) | | |
| | | | c | 8 | 900 (i) | | |
| | Standard 1, 2, 3 | 7 | a | 7 | 4500 (r) | 2x flushing | Washing the syringe and the $PO_4^{3-}$ detector with deionized water |
| | | | b | 8 | 4500 (i) | | |
| | | 8 | a | 13(14, 2)[d] | 4500 (r) | | |

| | | | | | | | |
|---|---|---|---|---|---|---|---|
| | | | b | 8 | 4500 (i) | 2x flushing | Washing the syringe and the $PO_4^{3-}$ detector with STD1 (STD2, STD3) |
| | | 9 | a | 13(14, 2) | 2700 (r) | | Draw up STD1(STD2, STD3) and set the light intensity for the reference $(I^R, V^R)$ |
| | | | b | 4 | 1350 (i) | | |
| | | | c | 8 | 1350 (i) | | |
| | | 10 | a | 8 | 720 (r) | | Withdraw STD1 from the flow cell and add R1 and R2 |
| | | | b | 9 | 180 (r) | | |
| | | | c | 10 | 180 (r) | | |
| | | 11 | a | 8 | 450 (i) / 450 (r) | 4x mixing | Mixing the reagent with STD1 and inject the reaction mixture inside the detector and waiting for 3 minutes for color development and set the light intensity for the measurement $(V,\ I)$ |
| | | | b | 4 | 180 (i) | | |
| | | | c | 8 | 900 (i) | | |
| washing | | 12 | a | 3 | 4500 (r) | | Draw up the cleaning solution into the syringe and all detectors, and set the light intensity for blank measurement. |
| | | | b | 8 | 4500 (i) | | |
| | | | c | 3 | 4500 (r) | | |
| | | | d | 15 | 4500 (i) | | |
| | | | e | 3 | 4500 (r) | | |
| | | | f | 16 | 4500 (i) | | |
| **Σ ($NO_3^-$ + $NO_2^-$) Calibration** | Blank | 13 | | 4 | 4500 (i) | | Initialize the syringe motor and detector, align the switching valve and ensure that the syringe plunger is fully retracted. |
| | | 14 | a | 7 | 4500 (r) | 2x flushing | Washing the syringe and the $PO_4^{3-}$ detector with deionized water |
| | | | b | 8 | 4500 (i) | | |
| | | 15 | a | 7 | 4500 (r) | 2x flushing | Washing the syringe and the Σ ($NO_3^-$ + $NO_2^-$) detector with deionized water |
| | | | b | 16 | 4500 (i) | | |
| | | 16 | a | 7 | 2700 (r) | | Draw up the blank and set the light intensity for the reference $(I^R, V^R)$ |
| | | | b | 4 | 700 (i) | | |
| | | | c | 16 | 2000 (i) | | |
| | | 17 | a | 16 | 1440 (r) | | Withdraw the blank from the flow cell and add mixed reagent (Griess reagent + $VCl_3$) |
| | | | b | 5 | 360 (r) | | |
| | | 18 | | 16 | 450 (i) / 450 (r) | 4x mixing | Mixing the blank with the reagent |
| | | 19 | | 8 | 1800 (i) | | Waiting 30 minutes into $PO_4^{3-}$ detector to allow $NO_3^-$ reduction during incubation with $VCl_3$ reagent at maximum temperature (~ 50 °C). |
| | | 20 | a | 8 | 1800 (r) | | Withdraw the reaction mixture from the $PO_4^{3-}$ detector, waiting 2 min. inside the syringe housing to cool the mixture and open the Σ ($NO_3^-$ + $NO_2^-$) detector, inject the mixture and set light intensity for the measurement $(V,\ I)$ |
| | | | b | 4 | 225 (i) | | |
| | | | c | 16 | 1575(i) | | |

| | | | | | | | |
|---|---|---|---|---|---|---|---|
| | Standard 1, 2, 3 | 21 | a | 7 | 4500 (r) | 2x flushing | Washing the syringe and the $PO_4^{3-}$ detector with deionized water |
| | | | b | 8 | 4500 (i) | | |
| | | 22 | a | 7 | 4500 (r) | 2x flushing | Washing the syringe and the Σ ($NO_3^-$ + $NO_2^-$) detector with deionized water |
| | | | b | 16 | 4500 (i) | | |
| | | 23 | a | 13(14, 2) | 4500 (r) | 2x flushing | Washing the syringe and the Σ ($NO_3^-$ + $NO_2^-$) detector with STD1 (STD2, STD3) |
| | | | b | 16 | 4500 (i) | | |
| | | 24 | a | 13(14, 2) | 2700 (r) | | Draw up STD1 (STD2, STD3) and set the light intensity for the reference ($I^R, V^R$) |
| | | | b | 4 | 700 (i) | | |
| | | | c | 16 | 2000 (i) | | |
| | | 25 | a | 16 | 1440 (r) | | Withdraw STD1 (STD2, STD3) from the flow cell and add mixed reagent (Griess reagent + $VCl_3$) |
| | | | b | 5 | 360 (r) | | |
| | | 26 | | 16 | 450 (i) | 4x mixing | Mixing STD1 (STD2, STD3) with the reagent |
| | | | | | 450 (r) | | |
| | | 27 | | 8 | 1800 (i) | | Waiting 30 minutes into $PO_4^{3-}$ detector to allow $NO_3^-$ reduction during incubation with $VCl_3$ reagent at maximum temperature (~ 50 °C). |
| | | 28 | a | 8 | 1800 (r) | | Withdraw the reaction mixture from the $PO_4^{3-}$ detector, waiting 2 min. inside the syringe housing to cool the mixture and open the Σ ($NO_3^-$ + $NO_2^-$) detector and inject the mixture and set light intensity for the measurement ($V$, $I$) |
| | | | b | 4 | 225 (i) | | |
| | | | c | 16 | 1575(i) | | |
| washing | | 29 | a | 3 | 4500 (r) | | Draw up the cleaning solution into the syringe and all detectors, and set the light intensity for blank measurement. |
| | | | b | 8 | 4500 (i) | | |
| | | | c | 3 | 4500 (r) | | |
| | | | d | 15 | 4500 (i) | | |
| | | | e | 3 | 4500 (r) | | |
| | | | f | 16 | 4500 (i) | | |
| **$H_4SiO_4$ Calibration** | | 30 | | 4 | 4500 (i) | | Initialize the syringe motor and detector, align the switching valve and ensure that the syringe plunger is fully retracted. |
| | Blank | 31 | a | 7 | 4500 (r) | 2x flushing | Washing the syringe and the $H_4SiO_4$ detector with deionized water |
| | | | b | 15 | 4500 (i) | | |
| | | 32 | a | 7 | 2700 (r) | | Draw up the blank and set the light intensity for the reference ($I^R, V^R$) |
| | | | b | 4 | 1350 (i) | | |
| | | | c | 15 | 1350 (i) | | |
| | | 33 | a | 15 | 225 (r) | | Withdraw the blank from the flow cell and add R1(molybdic acid) |
| | | | b | 11 | 225 (r) | | |
| | | 34 | | 15 | 300 (i) | 4x mixing | Mixing the blank and R1 and waiting 3 min for complexation |
| | | | | | 300 (r) | | |
| | | 35 | a | 12 | 225 (r) | | |

| | | | | | | | |
|---|---|---|---|---|---|---|---|
| | | | b | 10 | 225 (r) | | Add R2 (Oxalic acid) and R3 (Ascorbic Acid) to the reaction mixture |
| | | 36 | | 15 | 450 (i) | 4x mixing | Mixing the blank and the reagents |
| | | | | | 450 (r) | | |
| | | 37 | a | 4 | 225 (i) | | Inject the reaction mixture into the $H_4SiO_4$ detector and set the light intensity for measurement $(V,\ I)$ |
| | | | b | 15 | 675 (i) | | |
| | Standard 1, 2, 3 | 38 | a | 7 | 4500 (r) | 2x flushing | Washing the syringe and the $H_4SiO_4$ detector with deionized water |
| | | | b | 15 | 4500 (i) | | |
| | | 39 | a | 13(14, 2) | 4500 (r) | 2x flushing | Washing the syringe and the $H_4SiO_4$ detector with STD1 (STD2, STD3) |
| | | | b | 15 | 4500 (i) | | |
| | | 40 | a | 13(14, 2) | 2700 (r) | | Draw up STD1 (STD2, STD3) and set the light intensity for the reference $(I^R, V^R)$ |
| | | | b | 4 | 1350 (i) | | |
| | | | c | 15 | 1350 (i) | | |
| | | 41 | a | 15 | 225 (r) | | Withdraw STD1 (STD2, STD3) from the flow cell add R1(molybdic acid) |
| | | | b | 11 | 225 (r) | | |
| | | 42 | | 15 | 300 (i) | 4x mixing | Mixing STD1 (STD2, STD3) and R1 and waiting 3 min for complexation |
| | | | | | 300 (r) | | |
| | | 43 | a | 12 | 225 (r) | | Add R2 (Oxalic acid) and R3 (Ascorbic Acid) to the reaction mixture |
| | | | b | 10 | 225 (r) | | |
| | | 44 | | 15 | 450 (i) | 4x mixing | Mixing STD1 (STD2, STD3) and the reagents |
| | | | | | 450 (r) | | |
| | | 45 | a | 4 | 225 (i) | | Inject the reaction mixture into the $H_4SiO_4$ detector and set the light intensity for measurement $(V,\ I)$ |
| | | | b | 15 | 675 (i) | | |
| washing | | 46 | a | 3 | 4500 (r) | | Draw up the cleaning solution into the syringe and all detectors, and set the light intensity for blank measurement. |
| | | | b | 8 | 4500 (i) | | |
| | | | c | 3 | 4500 (r) | | |
| | | | d | 15 | 4500 (i) | | |
| | | | e | 3 | 4500 (r) | | |
| | | | f | 16 | 4500 (i) | | |
| **CRM** | $PO_4^{3-}$ determination | 47 | | 4 | 4500 (i) | | Initialize the syringe motor and detector, align the switching valve and ensure that the syringe plunger is fully retracted. |
| | | 48 | a | 7 | 4500 (r) | 2x flushing | Washing the syringe and the $PO_4^{3-}$ detector with deionized water |
| | | | b | 8 | 4500 (i) | | |
| | | 49 | | 6 | 4500 (r) | 2x flushing | Washing the syringe and the $PO_4^{3-}$ detector with CRM |
| | | | | 8 | 4500 (i) | | |
| | | 50 | a | 6 | 2700 (r) | | Draw up CRM and set the light intensity for the reference $(I^R, V^R)$ |
| | | | b | 4 | 1350 (i) | | |
| | | | c | 8 | 1350 (i) | | |
| | | 51 | a | 8 | 720 (r) | | Withdraw CRM from the flow cell and add R1 (Molybdate mixed reagent) and R2 (Ascorbic Acid) |
| | | | b | 9 | 180 (r) | | |

| | | | | | | | |
|---|---|---|---|---|---|---|---|
| | | | c | 10 | 180 (r) | | |
| | | 52 | | 8 | 450 (i) | 4x mixing | Mixing the reagent with CRM and inject the reaction mixture inside the detector and waiting for 3 minutes for color development and set the light intensity for the measurement $(V,\ I)$ |
| | | | | | 450 (r) | | |
| | | | | 4 | 180 (i) | | |
| | | | | 8 | 900 (i) | | |
| | Σ ($NO_3^-$ + $NO_2^-$) determination | 53 | a | 7 | 4500 (r) | 2x flushing | Washing the syringe and the $PO_4^{3-}$ detector with deionized water |
| | | | b | 8 | 4500 (i) | | |
| | | 54 | a | 6 | 4500 (r) | 2x flushing | Washing the syringe and the Σ ($NO_3^-$ + $NO_2^-$) detector with CRM |
| | | | b | 16 | 4500 (i) | | |
| | | 55 | a | 6 | 2700 (r) | | Draw up CRM and set the light intensity for the reference $(I^R, V^R)$ |
| | | | b | 4 | 700 (i) | | |
| | | | c | 16 | 2000 (i) | | |
| | | 56 | a | 16 | 1440 (r) | | Withdraw CRM from the flow cell and add mixed reagent (Griess reagent + $VCl_3$) |
| | | | b | 5 | 360 (r) | | |
| | | 57 | | 16 | 450 (i) | 4x mixing | Mixing CRM with the reagent |
| | | | | | 450 (r) | | |
| | | 58 | | 8 | 1800 (i) | | Waiting 30 minutes into $PO_4^{3-}$ detector to allow $NO_3^-$ reduction during incubation with $VCl_3$ reagent at maximum temperature (~ 50 °C). |
| | | 59 | a | 8 | 1800 (r) | | Withdraw the reaction mixture from the $PO_4^{3-}$ detector, waiting 2 min. inside the syringe housing to cool the mixture and open the Σ ($NO_3^-$ + $NO_2^-$) detector and inject the mixture and set light intensity for the measurement $(V,\ I)$ |
| | | | b | 4 | 225 (i) | | |
| | | | c | 16 | 1575 (i) | | |
| | **$H_4SiO_4$determination** | 60 | a | 7 | 4500 (r) | 2x flushing | Washing the syringe and the $H_4SiO_4$ detector with deionized water |
| | | | b | 15 | 4500 (i) | | |
| | | 61 | a | 6 | 4500 (r) | 2x flushing | Washing the syringe and the $H_4SiO_4$ detector with CRM |
| | | | b | 15 | 4500 (i) | | |
| | | 62 | a | 6 | 2700 (r) | | Draw up CRM and set the light intensity for the reference $(I^R, V^R)$ |
| | | | b | 4 | 1350 (i) | | |
| | | | c | 15 | 1350 (i) | | |
| | | 64 | a | 15 | 225 (r) | | Withdraw CRM from the flow cell and add R1(molybdic acid) |
| | | | b | 11 | 225 (r) | | |
| | | 65 | | 15 | 300 (i) | 4x mixing | Mixing CRM and R1 then waiting 3 min for complexation |
| | | | | | 300 (r) | | |
| | | 66 | a | 12 | 225 (r) | | Add R2 (Oxalic acid) and R3 (Ascorbic Acid) to the reaction mixture |
| | | | b | 10 | 225 (r) | | |
| | | 67 | | 15 | 450 (i) | 4x mixing | Mixing CRM and the reagents |
| | | | | | 450 (r) | | |
| | | 68 | a | 4 | 225 (i) | | |

| | | | | | | | | |
|---|---|---|---|---|---|---|---|---|
| | | | b | 15 | 675 (i) | | Inject the reaction mixture into the $H_4SiO_4$ detector and set the light intensity for measurement ($V$, $I$) | |
| washing | | 69 | a | 3 | 4500 (r) | | Draw up the cleaning solution into the syringe and all detectors, and set the light intensity for blank measurement. | |
| | | | b | 8 | 4500 (i) | | | |
| | | | c | 3 | 4500 (r) | | | |
| | | | d | 15 | 4500 (i) | | | |
| | | | e | 3 | 4500 (r) | | | |
| | | | f | 16 | 4500 (i) | | | |
| **Sea water** | $PO_4^{3-}$ determination | 70 | | 4 | 4500 (i) | | Initialize the syringe motor and detector, align the switching valve and ensure that the syringe plunger is fully retracted. | |
| | | 71 | a | 1 | 4500 (r) | 6x flushing | Washing the syringe and the $PO_4^{3-}$ detector with seawater | 10 x (measurement loop) |
| | | | b | 8 | 4500 (i) | | | |
| | | 72 | a | 1 | 2700 (r) | | Draw up seawater and set the light intensity for the reference ($I^R, V^R$) | |
| | | | b | 4 | 1350 (i) | | | |
| | | | c | 8 | 1350 (i) | | | |
| | | 73 | a | 8 | 720 (r) | | Withdraw seawater from the flow cell and add R1 (Molybdate mixed reagent) and R2 (Ascorbic Acid) | |
| | | | b | 9 | 180 (r) | | | |
| | | | c | 10 | 180 (r) | | | |
| | | 74 | a | 8 | 450 (i) | 4x mixing | Mixing the reagent with seawater and inject the reaction mixture inside the detector and waiting for 3 minutes for color development and set the light intensity for the measurement ($V$, $I$) | |
| | | | | | 450 (r) | | | |
| | | | b | 4 | 180 (i) | | | |
| | | | c | 8 | 900 (i) | | | |
| | Σ ($NO_3^-$ + $NO_2^-$) determination | 75 | a | 1 | 4500 (r) | 6x flushing | Washing the syringe and the Σ ($NO_3^-$ + $NO_2^-$) detector with seawater | |
| | | | b | 16 | 4500 (i) | | | |
| | | 76 | a | 1 | 2700 (r) | | Draw up seawater and set the light intensity for the reference ($I^R, V^R$) | |
| | | | b | 4 | 700 (i) | | | |
| | | | | 16 | 2000 (i) | | | |
| | | 77 | a | 16 | 1440 (r) | | Withdraw seawater from the flow cell and add mixed reagent (Griess reagent + $VCl_3$) | |
| | | | b | 5 | 360 (r) | | | |
| | | 78 | | 16 | 450 (i) | 4x mixing | Mixing seawater with the reagent | |
| | | | | | 450 (r) | | | |
| | | 79 | | 8 | 1800 (i) | | Waiting 30 minutes into $PO_4^{3-}$ detector to allow $NO_3^-$ reduction during incubation with $VCl_3$ reagent at maximum temperature (~ 50 °C). | |
| | | 80 | a | 8 | 1800 (r) | | Withdraw the reaction mixture from the $PO_4^{3-}$ detector, waiting 2 min. inside the syringe housing to cool the mixture and open the Σ ($NO_3^-$ + $NO_2^-$) detector and inject the mixture and set light intensity for the measurement ($V$, $I$) | |
| | | | b | 4 | 225 (i) | | | |
| | | | c | 16 | 1575 (i) | | | |

| | | | | | | | | |
|---|---|---|---|---|---|---|---|---|
| | **$H_4SiO_4$ determination** | 81 | a | 1 | 4500 (r) | 6x flushing | Washing the syringe and the $H_4SiO_4$ detector with seawater | |
| | | | b | 15 | 4500 (i) | | | |
| | | 82 | a | 1 | 2700 (r) | | Draw up seawater and set the light intensity for the reference ($I^R, V^R$) | |
| | | | b | 4 | 1350 (i) | | | |
| | | | c | 15 | 1350 (i) | | | |
| | | 83 | a | 15 | 225 (r) | | Withdraw seawater from the flow cell and add R1(molybdic acid) | |
| | | | b | 11 | 225 (r) | | | |
| | | 84 | | 15 | 300 (i) | 4x mixing | Mixing seawater and R1 then waiting 3 min for complexation | |
| | | | | | 300 (r) | | | |
| | | 85 | a | 12 | 225 (r) | | Add R2 (Oxalic acid) and R3 (Ascorbic Acid) to the reaction mixture | |
| | | | b | 10 | 225 (r) | | | |
| | | 86 | | 15 | 450 (i) | 4x mixing | Mixing seawater and the reagents | |
| | | | | | 450 (r) | | | |
| | | 87 | a | 4 | 225 (i) | | Inject the reaction mixture into the $H_4SiO_4$ detector and set the light intensity for measurement ($V$, $I$) | |
| | | | b | 15 | 675 (i) | | | |

[a] maximum movement of the syringe is 4500 (~2.2 ml)
[b] r: retract; [c] i: inject
[d] port 13: STD1, port 14: STD2, port 2: STD3

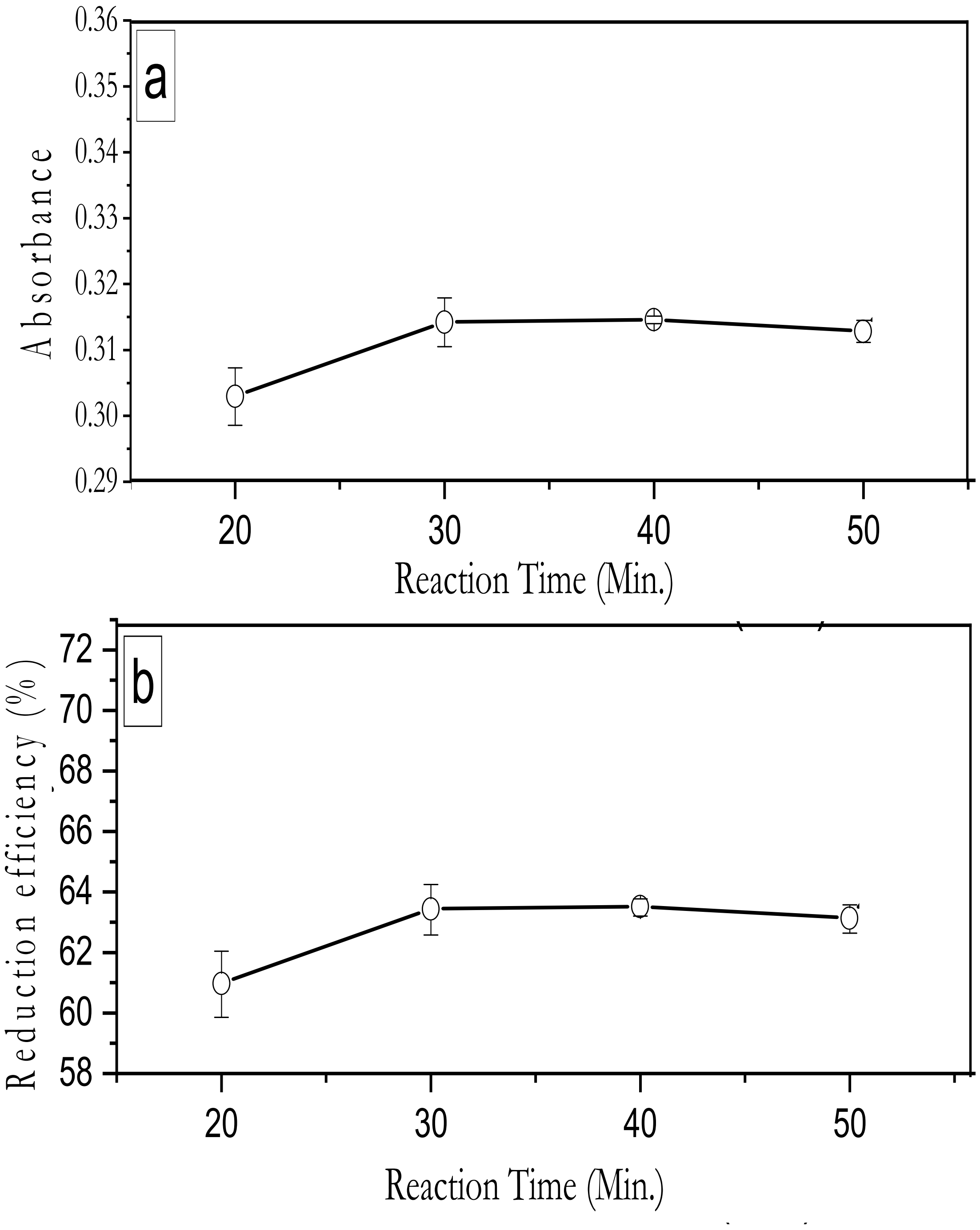


**Figure S1.** Effect of reaction time in minutes on (a) the absorbance of 10 μM $NO_3^-$ and (b) the reduction efficiency (%) which defined as the ratio of the absorbance of 10 μM $NO_3^-$ and the absorbance of 10 μM $NO_2^-$.

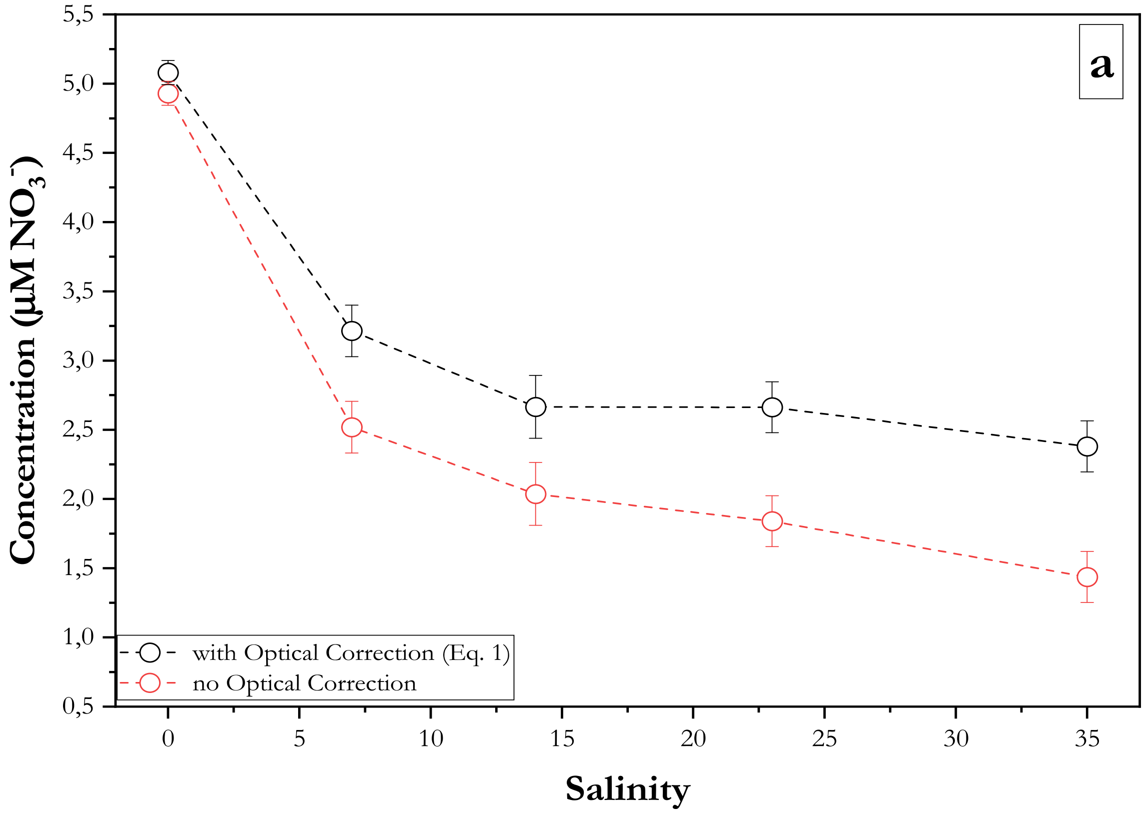
a
5,5
5,0
4,5
4,0
3,5
3,0
2,5
2,0
1,5
1,0
0,5
Concentration (µM NO3-)
0
5
10
15
20
25
30
35
Salinity
with Optical Correction (Eq. 1)
no Optical Correction

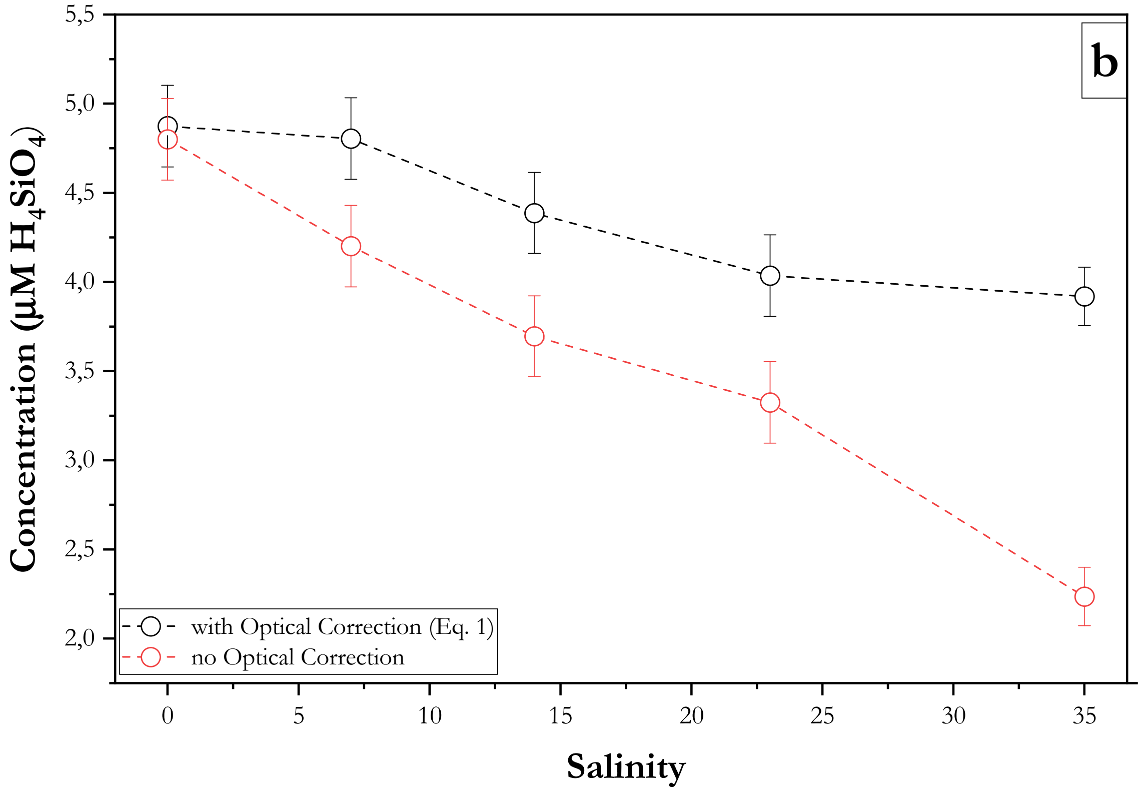
b
5,5
5,0
4,5
4,0
3,5
3,0
2,5
2,0
Concentration (µM H4SiO4)
0
5
10
15
20
25
30
35
Salinity
with Optical Correction (Eq. 1)
no Optical Correction

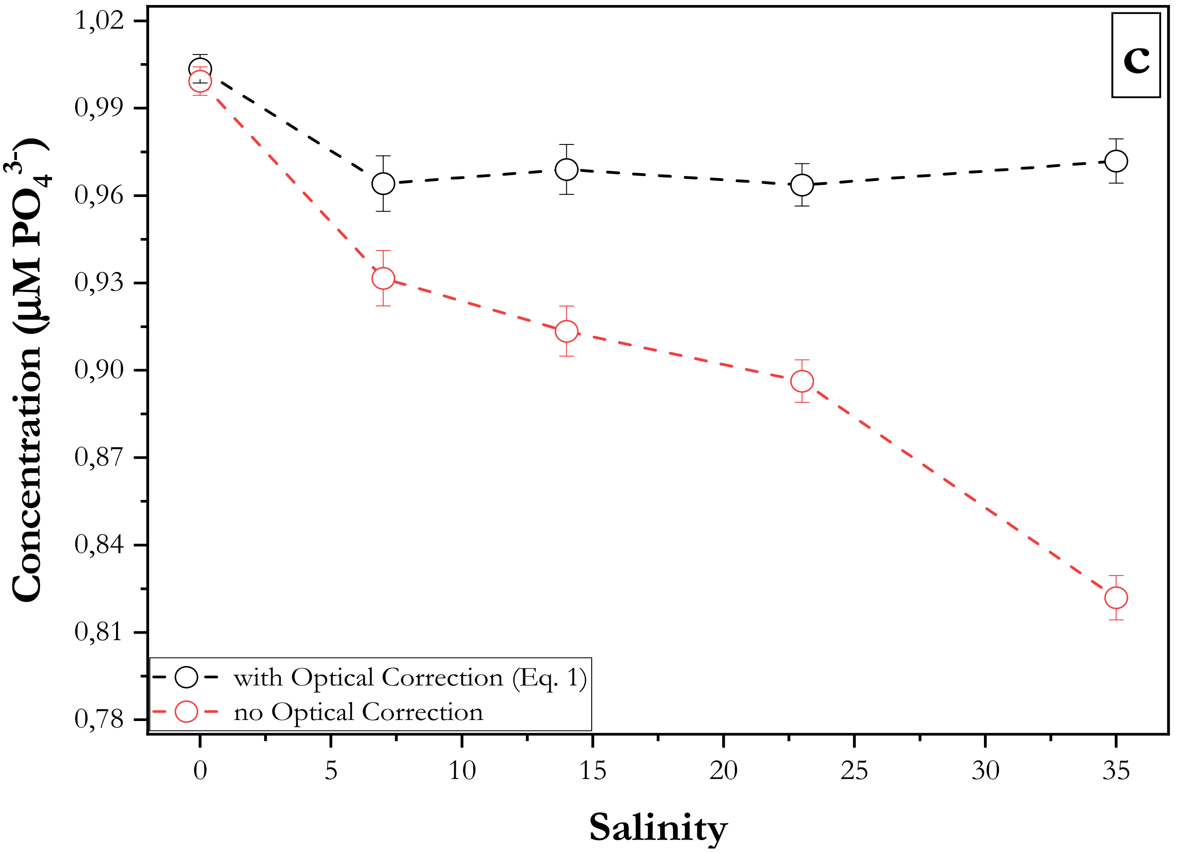


**Figure S2.** (a) calculated concentrations of 5 µM $NO_3^-$, (b) calculated concentrations of 5 µM $H_4O_4Si$ and (c) calculated concentrations of 1 µM $PO_4^{3-}$ samples with different salinity (0, 7, 14, 23, 35) based on calibration curves of (0, 1, 5 and 10 µM $NO_3^-$), (0, 1, 5, 10 µM $H_4O_4Si$) and (0, 0.5, 1, 2 µM $PO_4^{3-}$), respectively. The raw data were processed using equation 1 (black circles) and the red circles represent the data processed using the traditional Beer's Law equation ($A = -log_{10}(\frac{I}{I_0})$). Error bar (±1 SD), n = 10.

**Table S2.** Slopes and intercepts including their standard deviations, t-values and the probabilities for the calibration curves in **Figure 6**.

| | | Value | Standard deviation | t-value | Probability |
|---|---|---|---|---|---|
| $NO_3^-$ | **Int.** | 0.06876 | $6.85 \times 10^{-4}$ | 100.42 | $1.079 \times 10^{-13}$ |
| | **Slp.** | 0.00614 | $9.76 \times 10^{-5}$ | 62.94 | $4.51 \times 10^{-12}$ |
| $NO_2^-$ | **Int.** | 0.04061 | 0.00228 | 17.77 | $1.028 \times 10^{-7}$ |
| | **Slp.** | 0.01202 | $2.94 \times 10^{-4}$ | 40.87 | $1.414 \times 10^{-10}$ |
| $PO_4^{3-}$ | **Int.** | 0.00191 | $4.88 \times 10^{-4}$ | 3.9 | 0.00294 |
| | **Slp.** | 0.01211 | $8.46 \times 10^{-5}$ | 143.1 | $5 \times 10^{-10}$ |
| $H_4SiO_4$ | **Int.** | -0.00632 | 0.00351 | -1.8 | 0.10199 |
| | **Slp.** | 0.02377 | $6.48 \times 10^{-4}$ | 36.65 | $5.44 \times 10^{-12}$ |

Int.: Intercept, Slp. : Slope.

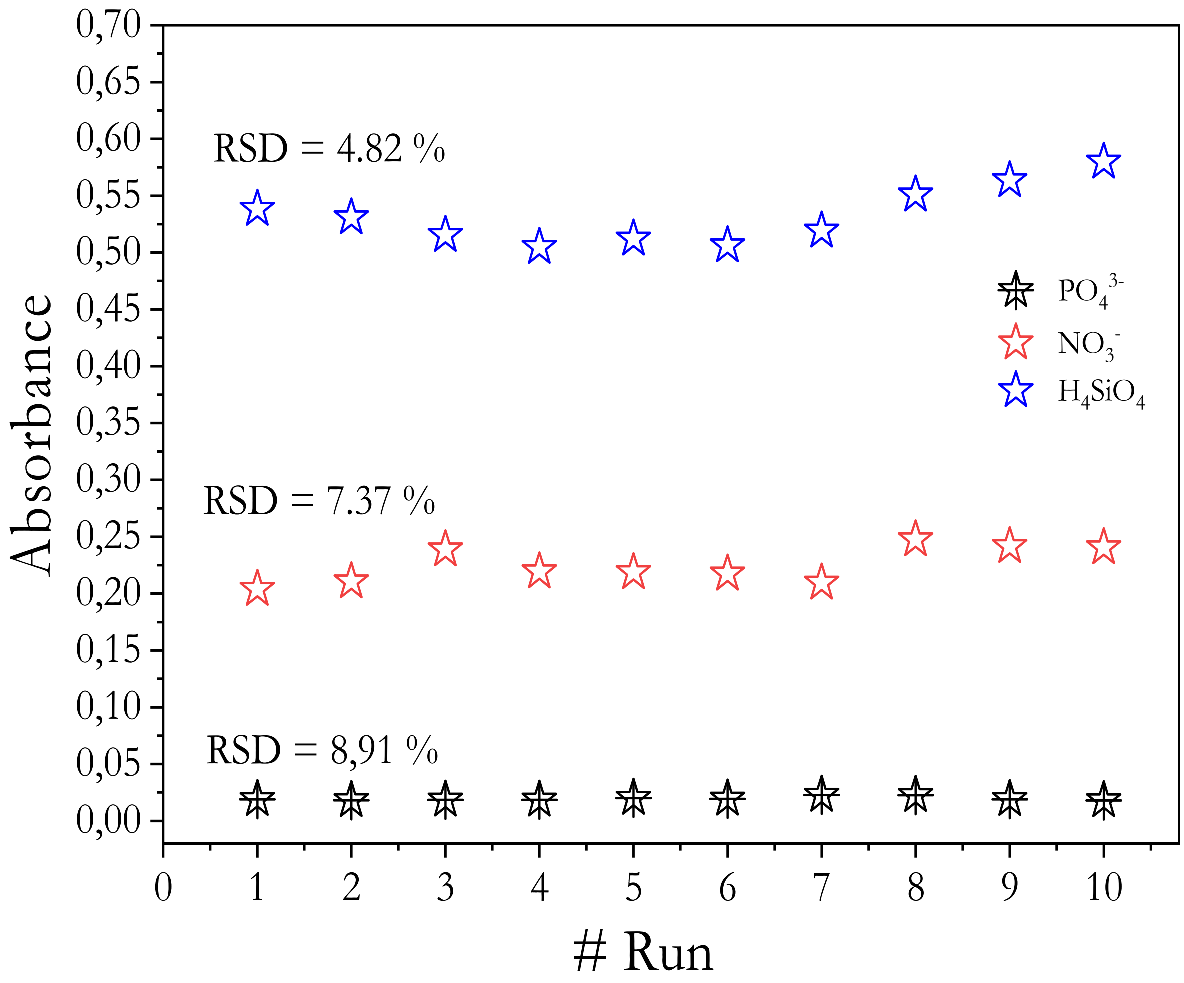


**Figure S3.** The measured absorbance value of KANSO CRM for nutrients for 6 consecutive runs of $PO_4^{3-}$, $NO_3^-$, and $H_4O_4Si$ with RSD (relative standard deviation) value. The Certified value for CRM is 23.7 ± 0.2 μM for $NO_3^-$, 56.4 ± 0.5 μM for $H_4O_4Si$, and 1.7 ± 0.02 μM for $PO_4^{3-}$.

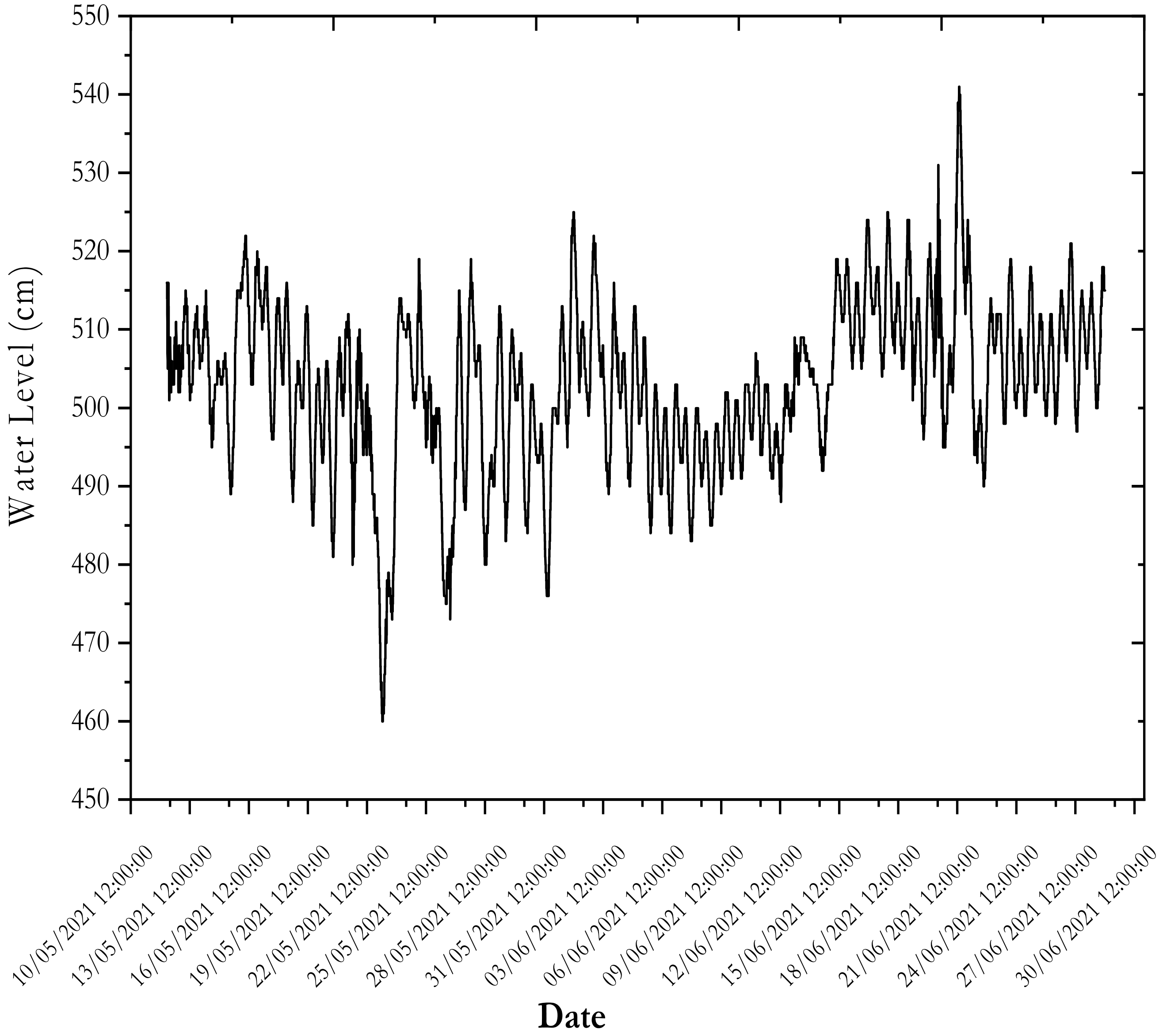


**Figure S4.** Time series data for the period from May 12 to June 28, 2021, for water level data at the kiel-Holtenau station obtained from the Federal Waterways and Shipping Administration (WSV).

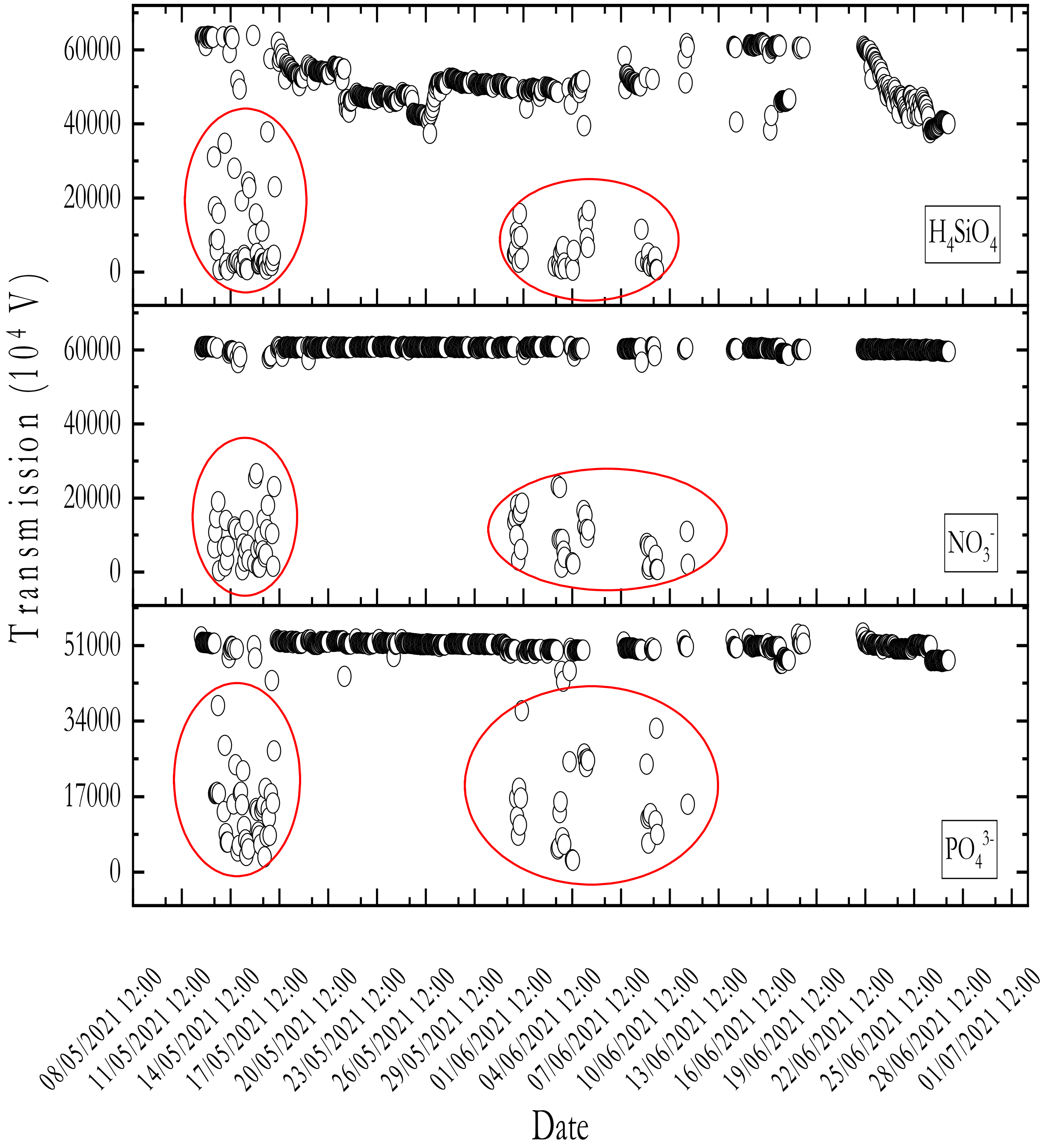


**Figure S5.** Time series data from May 12 to June 26, 2021, of $PO_4^{3-}$, $NO_3^-$, and $H_4O_4Si$ photodiode detector readout; the points in red circles refer to the drop-down of the transmission values due to air bubbles trapped into the flow cell.

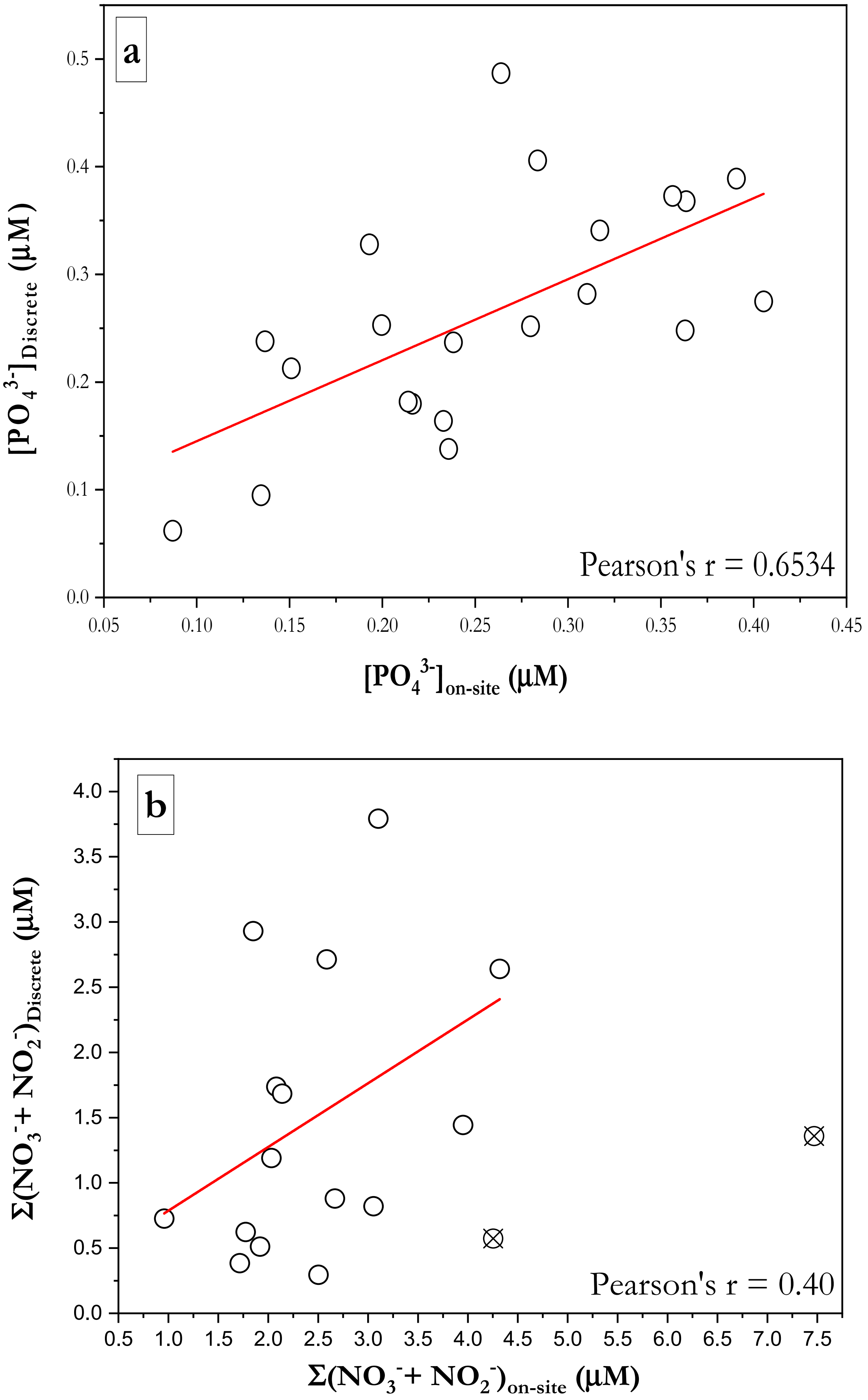

a
Pearson's r = 0.6534
0.0
0.1
0.2
0.3
0.4
0.5
0.05
0.10
0.15
0.20
0.25
0.30
0.35
0.40
0.45
$[PO_4^{3-}]_{Discrete}$ (μM)
$[PO_4^{3-}]_{on\text{-}site}$ (μM)
b
Pearson's r = 0.40
0.0
0.5
1.0
1.5
2.0
2.5
3.0
3.5
4.0
0.5
1.0
1.5
2.0
2.5
3.0
3.5
4.0
4.5
5.0
5.5
6.0
6.5
7.0
7.5
$\Sigma(NO_3^- + NO_2^-)_{Discrete}$ (μM)
$\Sigma(NO_3^- + NO_2^-)_{on\text{-}site}$ (μM)

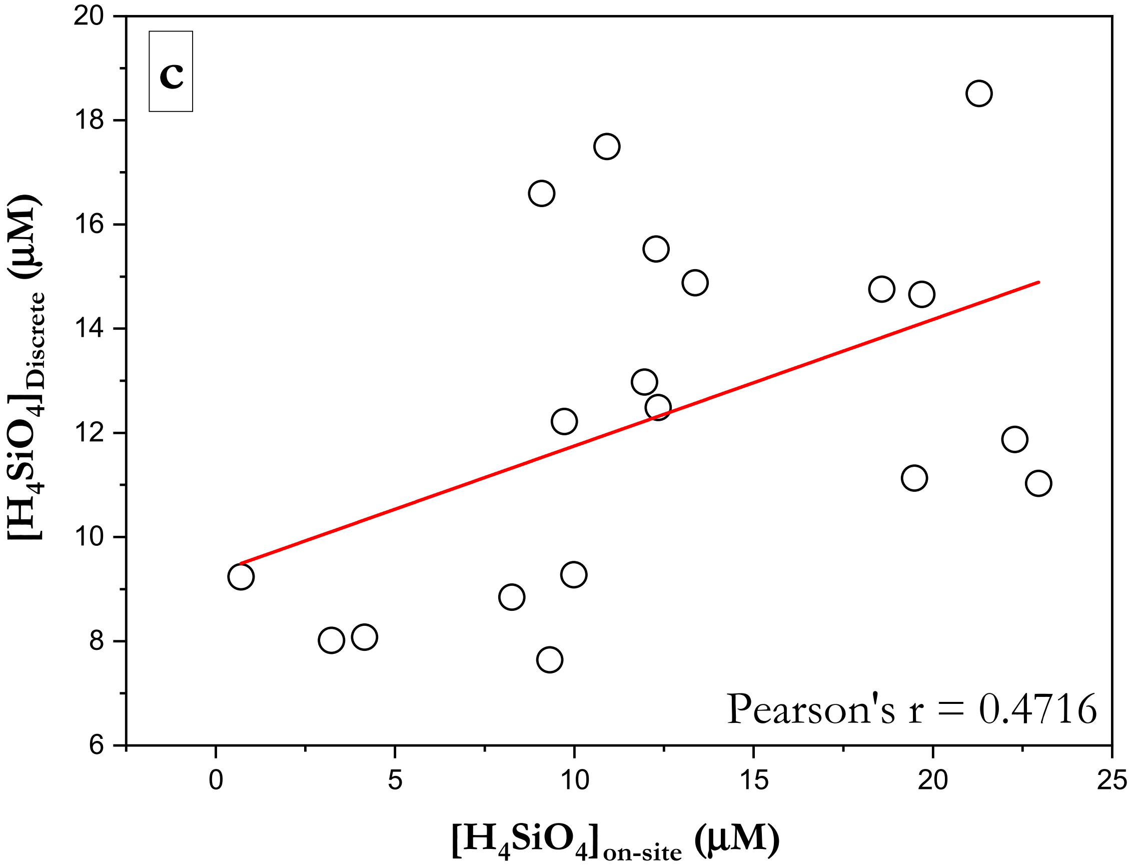


**Figure S6.** Property-to-property plots for (a) $PO_4^{3-}$ in µM measured on-site with the AutoLAB analyser compared to synchronised $PO_4^{3-}$ in µM measured with the air-segment analyser in the laboratory for discretely collected samples pearson'r = 0.6534, n = 21 , (b) $\Sigma(NO_3^- + NO_2^-)$ in µM measured on-site with the AutoLAB analyser compared to synchronised $\Sigma(NO_3^- + NO_2^-)$ in µM with the air-segment analyser in the laboratory for discretely collected samples, pearson'r = 0.4, n =17, two clear outliers (×) were removed and (c) $H_4SiO_4$ in µM measured on-site with the AutoLAB analyser compared to synchronised $H_4SiO_4$ in µM measured with the air-segment analyser in the laboratory for discretely collected samples, pearson'r = 0.4716, n = 19.

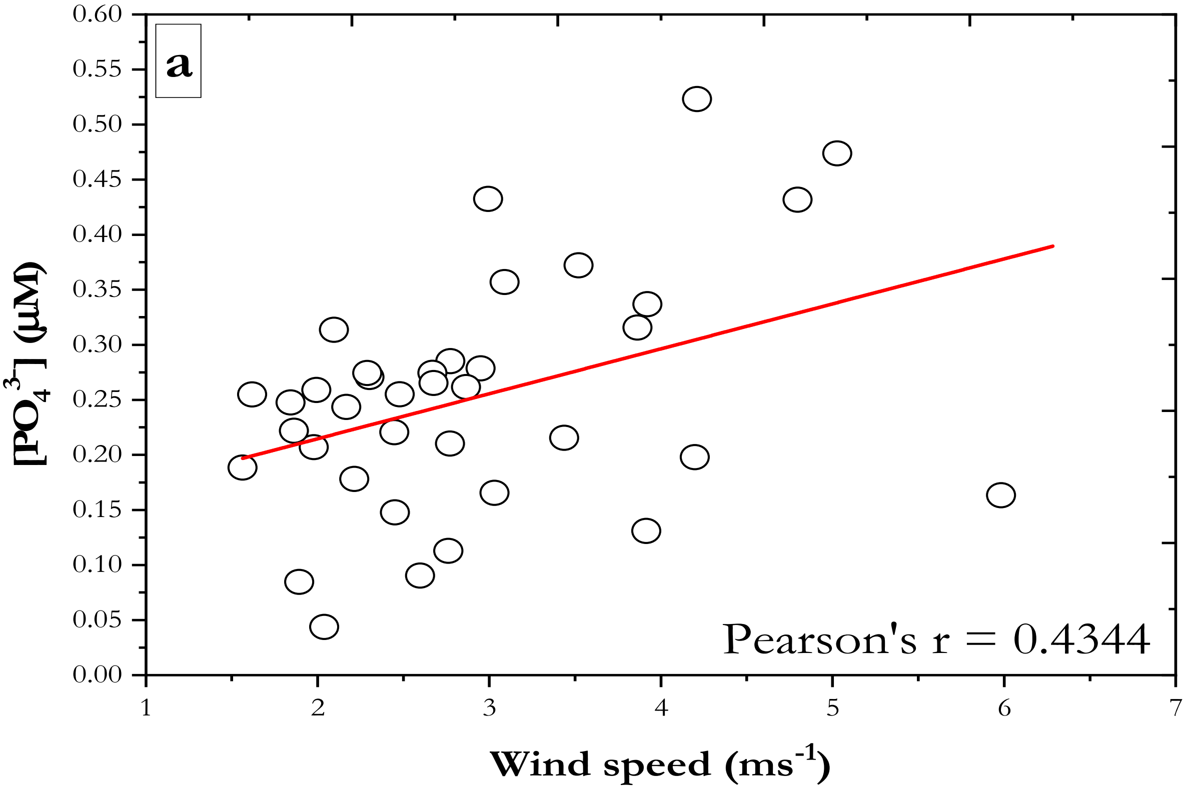
a
Pearson's r = 0.4344
[PO4 3-] (μM)
Wind speed (ms-1)

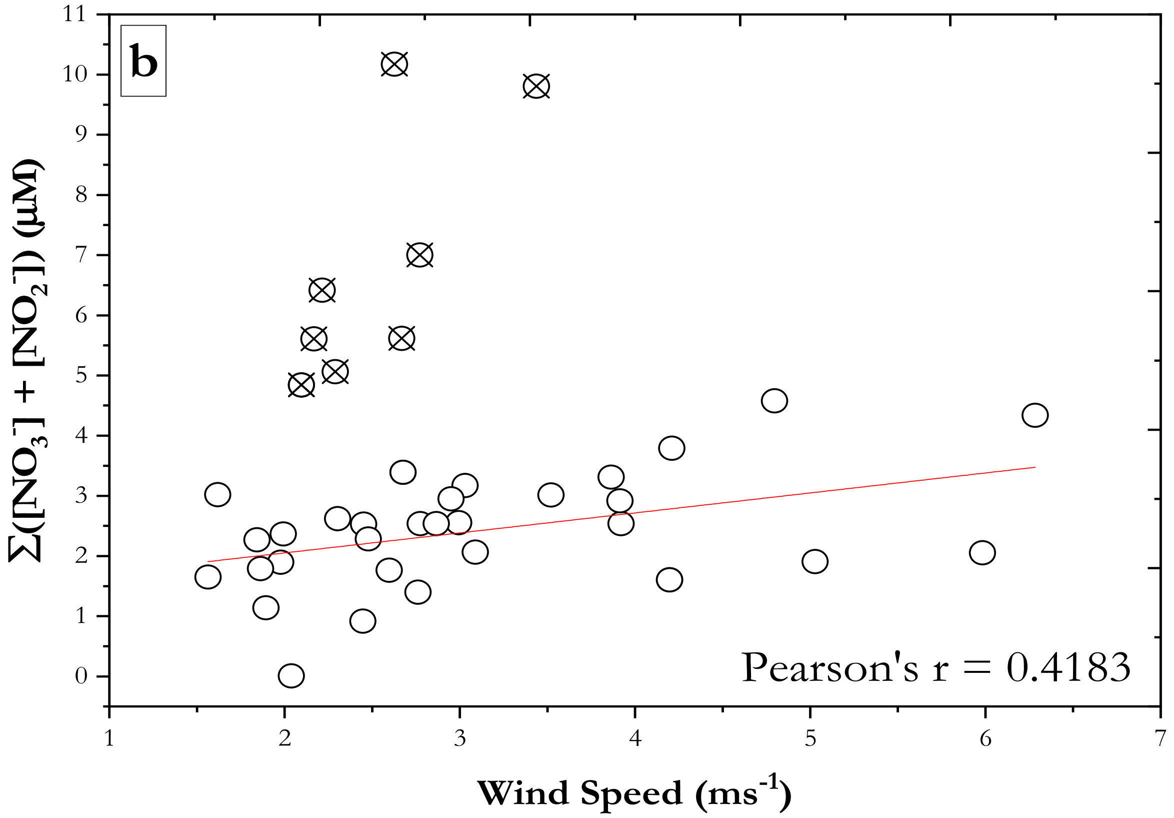
b
Pearson's r = 0.4183
Σ([NO3-] + [NO2-]) (μM)
Wind Speed (ms-1)

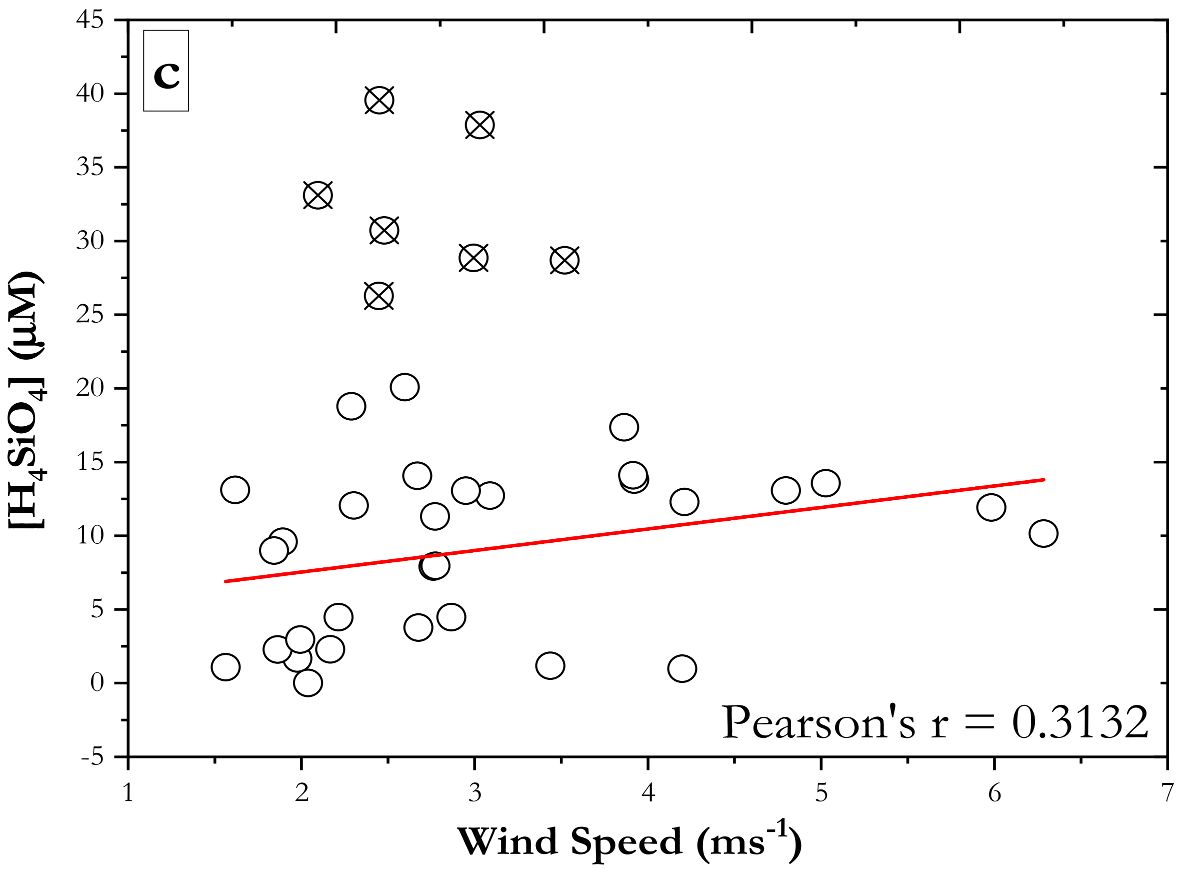


**Figure S7.** Plot-by-plot plots from 12 May to 27 June 2021 for (a) the daily average of on-site $PO_4^{3-}$ concentration in μM versus the daily average of wind speed in $ms^{-1}$, (b) the daily average of on-site $\Sigma(NO_3^- + NO_2^-)$ concentration in μM versus the daily average of wind speed in ms-1. the daily average of wind speed in ms-1, excluding clear outliers (9 points) (×), and (c) the daily average of $H_4SiO_4$ concentration in μM measured on site compared to the daily average of wind speed in $ms^{-1}$, excluding clear outliers (7 points) (×).

.

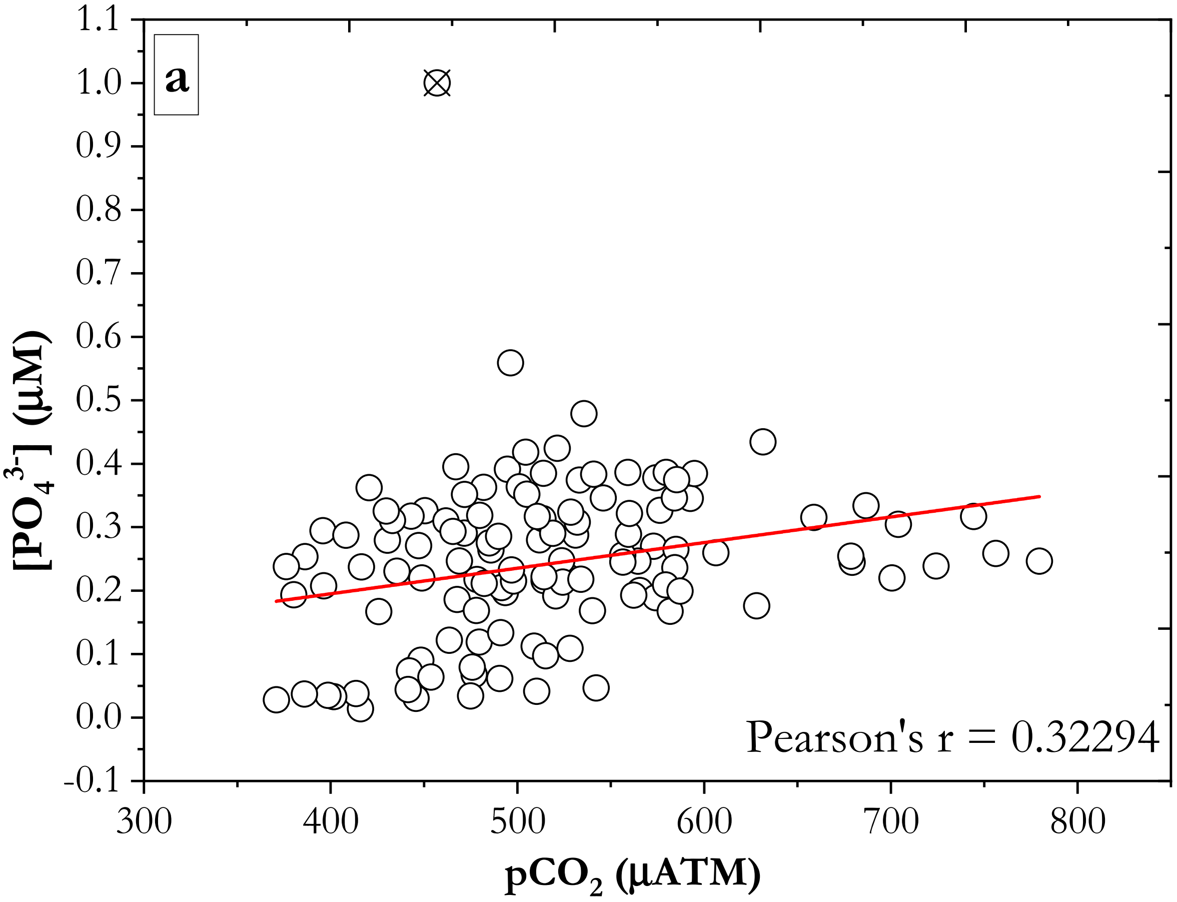
a
Pearson's r = 0.32294
[PO4 3-] (μM)
pCO2 (μATM)
1.1
1.0
0.9
0.8
0.7
0.6
0.5
0.4
0.3
0.2
0.1
0.0
-0.1
300
400
500
600
700
800

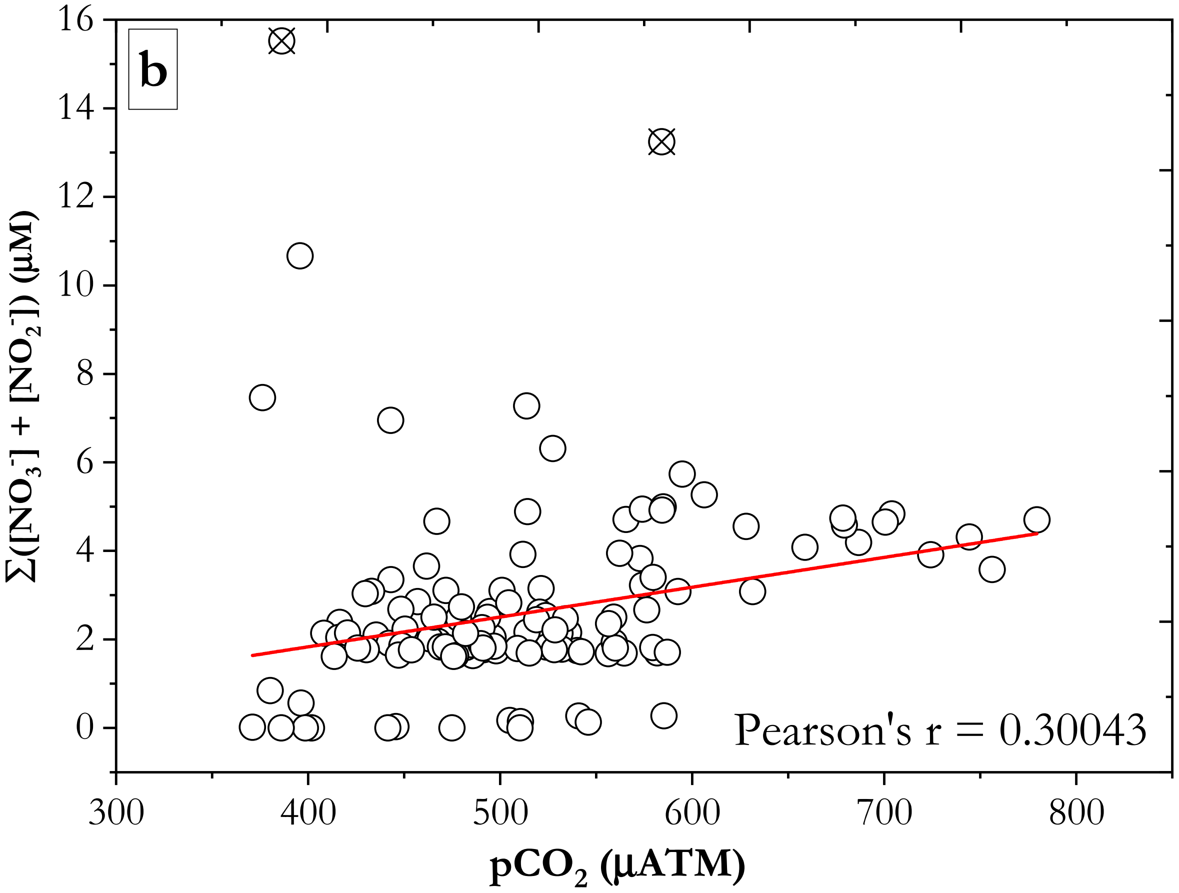
b
Pearson's r = 0.30043
Σ([NO3-] + [NO2-]) (µM)
pCO2 (µATM)
0
2
4
6
8
10
12
14
16
300
400
500
600
700
800

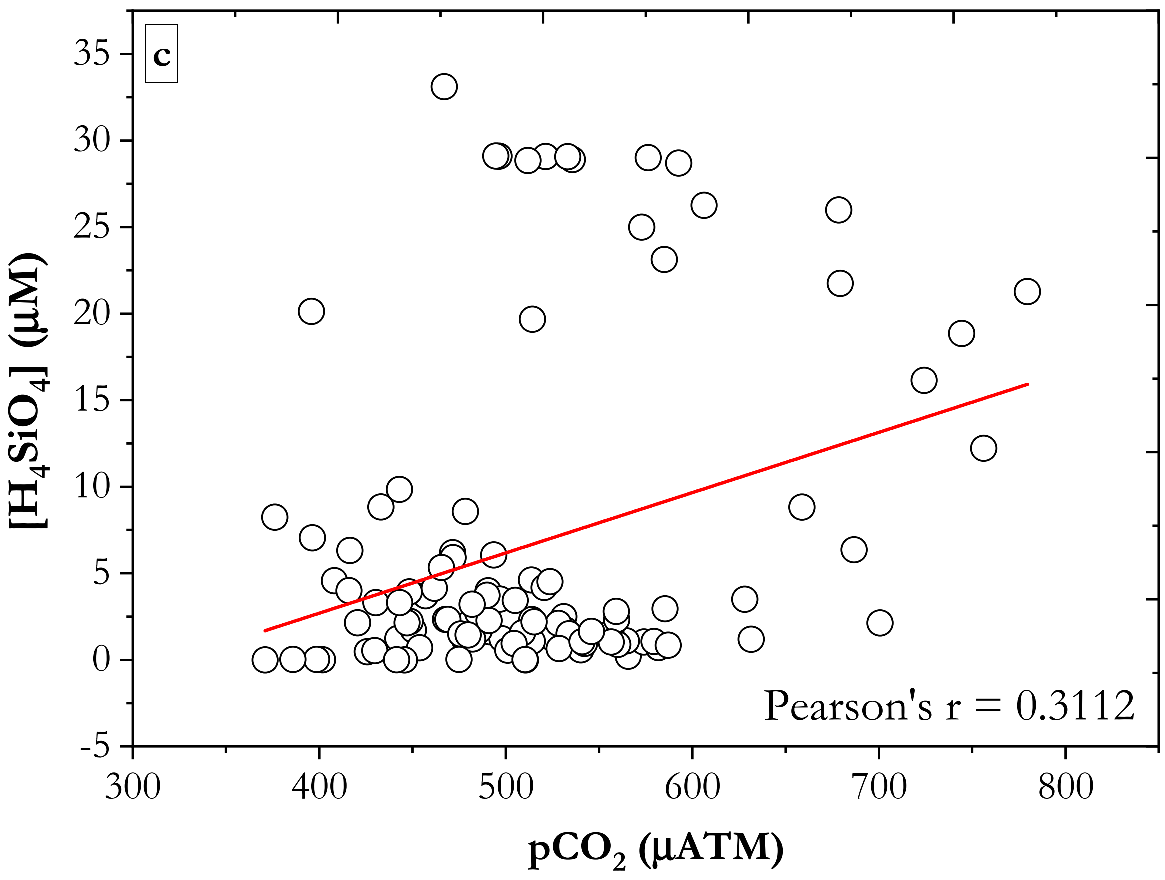


**Figure S8.** Plot-by-plot plots for the 11-day period from June 4 to June 9 and from June 18 and June 22 to June 27, 2021 for (a) in situ $pCO_2$ data compared to on-site $PO_4^{3-}$ measured by AutoLab with a unique outlier (×) was excluded (pearson'r = 0.32294, n = 122), (b) in situ $pCO_2$ data compared with on site $\Sigma(NO_3^- + NO_2^-)$ measured by AutoLab with two clear outliers (×) excluded (pearson'r = 0.30034, n = 122), and (c) in situ $pCO_2$ data compared with on site $H_4SiO_4$ measured by AutoLab (pearson'r = 0.3112, n = 108).
